# Lichen Striatus with Nail Involvement in a 6-Year-Old Boy

**DOI:** 10.1155/2020/1494760

**Published:** 2020-01-27

**Authors:** Alexander K. C. Leung, Kin Fon Leong, Benjamin Barankin

**Affiliations:** ^1^Department of Pediatrics, University of Calgary, Calgary, Alberta T2M 0H5, Canada; ^2^Alberta Children's Hospital, Calgary, Alberta T2M 0H5, Canada; ^3^Pediatric Institute, Kuala Lumpur General Hospital, Kuala Lumpur, Malaysia; ^4^Toronto Dermatology Centre, Toronto, Ontario M3H 5Y8, Canada

## Abstract

We describe a 6-year-old boy with an asymptomatic linear eruption on the left index finger with mild erythema of the proximal nail fold, nail dystrophy, and subungual hyperkeratosis of the nail. A diagnosis of nail lichen striatus was made. The child was successfully treated with a topical corticosteroid. Because of its rarity, nail lichen striatus is often under-recognized. Physicians should be familiar with the nail involvement in individuals with lichen striatus so that an accurate diagnosis can be made and unnecessary investigations and treatment avoided.

## 1. Introduction

Nail lichen striatus is rare [1]. There are approximately 30 cases of nail lichen striatus reported in the literature [1]. The condition is likely under-reported and under-recognized partly due to the failure to recognize the nail changes associated with lichen striatus. There is no established treatment of nail lichen striatus. Herein, we report a 6-year-old boy with nail lichen striatus who responded to topical mometasone cream twice a day for three months to the skin lesions and proximal nail fold with resolution of the skin lesions and onychodystrophy.

## 2. Case Report

A 6-year-old boy presented with a 10-month history of an asymptomatic linear eruption on the left index finger which gradually extended to the periungual area with resulting nail changes. His past health was unremarkable. There was no history of trauma for the affected areas. The family history was unremarkable.

**Figure 1:**
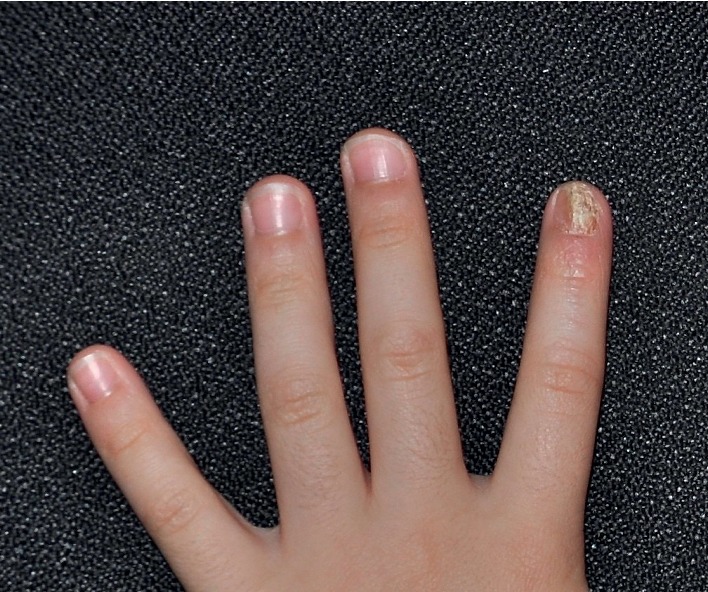
Multiple minute skin-colored papules in a linear distribution on the left index finger, mild erythema of the proximal nail fold, and onychodystrophy (hard to appreciate ridging in the image) and subungual hyperkeratosis in the nail.

**Figure 2:**
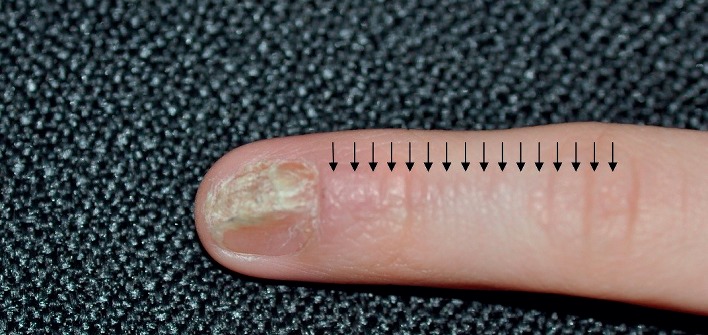
Close-up view of the left index finger showing multiple minute skin-colored papules (indicated by arrows) in a linear distribution, mild erythema of the proximal nail fold, and onychodystrophy and subungual hyperkeratosis in the nail.

**Figure 3:**
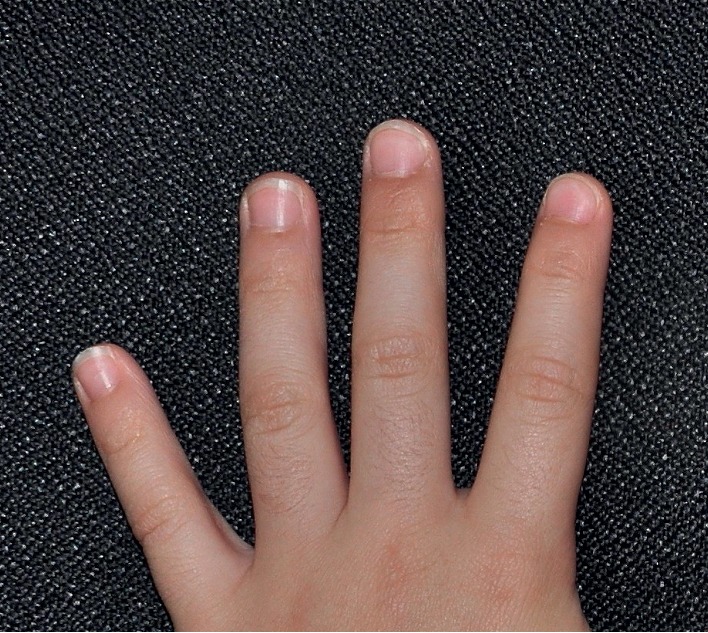
Complete resolution of nail lesions on the left index finger at one-year follow-up.

Physical examination revealed multiple tiny skin-colored papules along Blaschko lines on the left index finger, mild erythema of the proximal nail fold, and onychodystrophy and subungual hyperkeratosis of the nail (Figures 1 and 2). The rest of the physical examination was normal. Based on the characteristic physical findings, a diagnosis of nail lichen striatus was made. The child was treated with topical mometasone cream twice a day for three months over the skin lesions and liberal use of emollients. The skin lesions resolved in three months. At one-year follow-up, no onychodystrophy was noted (Figure 3). According to the parents, the nail lesions had resolved four months prior to the one-year scheduled follow-up.

## 3. Discussion

Lichen striatus is a benign, acquired, asymptomatic, self-limited T-cell-mediated dermatosis that primarily occurs in children between 4 months and 15 years of age [1, 2]. The male to female ratio is approximately 1 : 2 [2]. Lichen striatus is characterized by an abrupt onset of discrete, asymptomatic, flesh-colored, pink, tan, or erythematous, small (<3 mm), flat-topped papules that follow Blaschko lines [3]. Papules often coalesce to form a hyperpigmented continuous or interrupted linear band over a few weeks [3, 4]. The linear band, usually a few cm long and a few mm to 2 cm wide, may develop a curved appearance as it typically follows Blaschko lines [4]. The papules may appear hypopigmented (lichen striatus albus) in dark-skinned individuals [3, 4]. Typically, the eruption is solitary and unilateral [4]. V-shaped lines on the mid-back may be bilateral. Although lichen striatus may involve any parts of the body, the extremities are most commonly affected, followed by the trunk, buttocks, face, and neck [2, 4]. Typically, the lesion starts on a proximal extremity and extends distally [2]. Pruritus is uncommon [2].

Involvement of the nail is rare and may occur before, after, or concurrent with the skin lesion of lichen striatus [1–3, 5]. Very rarely, isolated lichen striatus limited to the nail unit without cutaneous eruptions has been reported [1, 6]. T-cell-mediated inflammation of the nail matrix leading to defective keratin synthesis in the nail plate is believed to be responsible for the onychodystrophy [1, 2, 5]. Onychodystrophy may take the form of nail pitting, longitudinal ridging (onychorrhexis), fissuring, splitting, fraying, pitting, onycholysis, striate or punctate leukonychia, subungual hyperkeratosis, overcurvature of the nail plate, thinning of the nail plate, thickening of the nail plate (onychauxis), irregular transverse grooves, and, rarely, nail loss [7–10]. Usually, only the lateral or medial portion of the nail plate of only one nail is affected, as is illustrated in the present case [3].

In the present case, a diagnosis of nail lichen striatus was made based on onychodystrophy and subungual hyperkeratosis localized to one portion of the nail, single nail involvement, and presence of typical skin lesions of lichen striatus near the nail. Lichen striatus should be differentiated from cutaneous lichen planus and inflammatory linear verrucous epidermal nevus (ILVEN). Cutaneous lichen planus is characterized by 6 Ps: planar (flat-topped), purple (violaceous), polygonal, pruritic, papules/plaques that affect the skin. Lesions of lichen planus are often superimposed by lacy, reticular, white lines known as “Wickham striae.” Linear lichen planus is a distinct variant of lichen planus with papules in a linear distribution that may mimic lichen striatus. ILVEN is characterized by intensely pruritic, skin-colored to brown, hyperkeratotic papules linearly distributed along lines of Blaschko. The condition usually presents at birth or shortly thereafter, progresses slowly, and tends to be persistent. ILVEN often involves the lower limb or buttock and is usually unilateral.

Because of the rarity of cases, there is no established treatment of nail lichen striatus. Some authors suggest watchful observation due to the benign and self-limited nature of nail lichen striatus [10]. Other authors suggest the use of topical corticosteroid or topical immunomodulator (tacrolimus or pimecrolimus) over the skin lesion or intralesional steroid injection in the proximal nail fold to hasten the resolution of nail lichen striatus [6, 8]. Generally, onychodystrophy subsides spontaneously with resolution of the skin lesions of lichen striatus [10]. In the present case, our patient responded to three months' topical corticosteroid treatment with resolution of skin lesions of lichen striatus and, subsequently, onychodystrophy. Thus, intralesional steroid injection in the proximal nail fold might not be necessary.

## 4. Conclusion

We describe a 6-year-old boy with nail lichen striatus who was successfully treated with a topical corticosteroid. Thus far, there is no established treatment of nail lichen striatus. This case serves as an example that the nail lichen striatus can be successfully treated with a topical corticosteroid. Because of its rarity, nail lichen striatus is often under-recognized. Physicians should be familiar with the nail involvement of lichen striatus so that an accurate diagnosis can be made and unnecessary investigations and inappropriate treatment avoided.
